# Author self-citation in orthodontics is associated with author origin and gender

**DOI:** 10.1186/s40510-020-00348-y

**Published:** 2021-01-07

**Authors:** Christos Livas, Konstantina Delli, Nikolaos Pandis

**Affiliations:** 1 Dental Clinics Zwolle, Zwolle, The Netherlands; 2 Department of Oral and Maxillofacial Surgery, University of Groningen, University Medical Center Groningen, Groningen, The Netherlands; 3 Department of Orthodontics and Dentofacial Orthopaedics, School of Dental Medicine, University of Bern, Bern, Switzerland

**Keywords:** Bibliometrics, Author self-citation, Impact factor, Orthodontic journals

## Abstract

**Background:**

The aims of this bibliometric study were to determine author self-citation trends in high-impact orthodontic literature and to investigate possible association between self-citation and publication characteristics.

**Methods:**

Six orthodontic journals with the highest impact factor as ranked by 2017 Journal Citation Reports were screened for a full publication year (2018) for original research articles, reviews, and case reports. Eligible articles were scrutinized for article and author characteristics and citation metrics. Univariable and multivariable negative binomial regression was used to examine associations between self-citation incidence and publication characteristics.

**Results:**

Medians for author self-citation rate of the most self-citing authors and self-citations were 3.03% (range 0–50) and 1 (range 0–19), respectively. In the univariable analysis, there was no association between self-citation counts and study type (*P* = 0.41), article topic (*P* = 0.61), number of authors (*P* = 0.62), and rank of authors (*P* = 0.56). Author origin (*P* = 0.001), gender (*P* = 0.001) and journal (*P* = 0.05) were associated with self-citation counts and in the multivariable analysis only origin and gender remained strong self-citation predictors. Asian authors and females self-cited significantly less often than all other regions and male authors.

**Conclusions:**

Authors in orthodontics do not self-cite at a frequency that suggests potential citation manipulation. Author origin and gender were the only variables associated with citations counts. More bibliometric research is necessary to draw solid conclusions about author self-citation trends in orthodontic literature.

**Supplementary Information:**

The online version contains supplementary material available at 10.1186/s40510-020-00348-y.

## Background

In the modern academic world, scholar productivity determines to a large extent ranking of institutions, academic career promotion, and funding [1]. To measure the cumulative impact and relevance of the scientific research output of individual researchers, author-level metrics like citation counts and h-index are being regularly reviewed by tenure-track and grant advisory committees. Essentially, an author’s h-index captures the number of his/her publications that received at least the same number of citations including author self-citations, that is references to own previous work [2].

Self-citation has been labeled as “the hallmark of productive authors” since the more articles one has self-authored, the higher the probability for self-citation [3]. Genuine self-citation allows authors to expand previous research hypotheses, replicate established methodology, and rationalize the endorsement of new studies [4]. On the other hand, unethical practices such as excessive and superfluous self-citation have been criticized for artificially inflating citation-based metrics and self-promotion [5].

A small number of studies has investigated author self-citation in medical literature [4, 6, 7]. Almost one fifth of all citations to articles about diabetes mellitus in 2000 derived directly from their authors [4]. Author self-citations accounted for 6.5% of the total citations referred to articles published in high-profile general medicine journals [6]. Self-citations represented 9.5% of total citations in otolaryngology journals published in a 3-month period [7].

Bibliometric research in dentistry has studied so far self-citation merely at the journal level, revealing relatively low rates, and therefore favorable publishing conditions and citation behaviors [8–10]. Given the ethical implications and lack of evidence in orthodontics, this study aimed to determine author self-citation rates in highly esteemed orthodontic journals. Furthermore, it intended to investigate author self-citation patterns in relation to article and author characteristics.

## Methods

### Data collection

The top 6 impact factor (IF) orthodontic journals as listed in 2017 Journal Citation Reports (JCR, Clarivate Analytics, Philadelphia, PA, USA) were examined in this study, namely *Orthodontics & Craniofacial research* (OCR; IF, 2.077), *European Journal of Orthodontics*, (EJO; IF, 2.033), *American Journal of Orthodontics and Dentofacial Orthopedics* (AJODO; IF, 1.842), *The Korean Journal of Orthodontics* (KJO; IF, 1.617), *The Angle Orthodontist* (AO; IF, 1.592), and *Progress in Orthodontics* (PIOR; IF, 1.250).

All issues published by the abovementioned journals between January and December 2018 were accessed using institutional subscription and hand-searched for original research articles, reviews, and case reports. Articles not falling into these categories were excluded.

Two investigators (first and second authors) underwent a 4-h training in article screening and data extraction, divided in 2 sessions. In the first session, each investigator screened titles and abstracts of a preselected sample of 40 articles. Any practical issues encountered were discussed during the second session. For the purposes of the study, the following information was extracted simultaneously and on a consensus basis from each eligible article: (i) journal title, (ii) article title, (iii) names of first and last authors, (iv) study type, i.e., randomized clinical trials, prospective observational study, retrospective observational study, narrative review, systematic review or meta-analysis, survey, case report [6], (v) topic, i.e., oral health-related quality of life (OHRQOL)/esthetics/practice management/socio-demographics, biomaterials, diagnosis, treatment, growth, new technologies, periodontics/caries prevention, side effects, other [1], (vi) number of authors, (vii) number of total citations (TC), (viii) number of self-citations, (ix) self-citation rate (SCR) calculated as the percentage of the author self-citations to the total citations included in the reference list, also termed as synchronous self-citation [11], (x) author rank, i.e., first, last or first/last in case of an equal number of self-citations assigned to both first and last authors, (xi) gender, and (xii) origin as indicated by the geographical location of the affiliation of the most self-citing author (first or last author). To facilitate data analysis, regions were classified into 5 groups, i.e., Asia, Europe, North America, South America, and other, which included Africa, Oceania, or a combination of continents. Articles were grouped as well according to the number of authors as follows: 1–3, 4–5, and > 5 authors. Gender of the authors was determined using genderize.io (https://genderize.io/; Demografix ApS, Roskilde, Denmark), a free online service that collects data from social networks across 79 countries and 89 languages. This tool was chosen due to its superior performance in gender prediction compared to other name-to-gender inference services [12]. The collected data were entered into a Microsoft Excel spreadsheet (Microsoft Corporation, Redmond, VA, USA) for further processing.

### Statistical analysis

Descriptive statistics were calculated for self-citations per predictor, i.e., journal, study type, article topic, number of authors, rank, origin, and gender. Given the presence of overdispersion, univariable negative binomial regression was used to examine potential associations between self-citations and the article and author characteristics. Significant predictors from the first step were added in multivariable negative binomial model. Overall, significance per predictor was assessed using the likelihood ratio test. All analyses were conducted with Stata 16.1 (Stata Corp, TX, USA) and R Software version 3.6.1 (R Foundation for Statistical Computing, Vienna, Austria) with a two-sided 5% level of statistical significance.

## Results

**Fig. 1:**
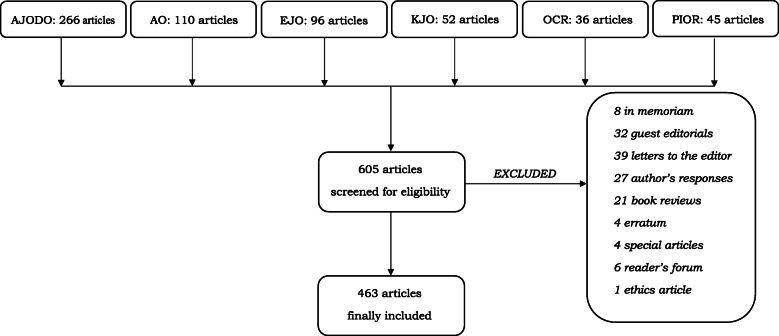
Flowchart of the selection process

By searching 2018 journal issues, 605 articles were initially identified. Screening for eligibility led to exclusion of 142 irrelevant articles, which were mainly classified as letters to the editor, guest editorials, and author’s responses (Fig. 1). Following a strict selection process, a total of 463 unique articles were included in the study.

AJODO contributed most of the articles (35.64%), while prospective observational (195 articles) and retrospective observational studies (117 articles) represented most of the articles. The most popular topics were treatment, diagnosis, and OHRQOL/esthetics/practice management/socio-demographics, investigated in 27.43%, 18.79%, and 13.39% of the articles, respectively (Table 1).

**Table 1:** Distribution of articles per study type, topic, number of authors, presence of self-citations, and descriptive statistics of citation metrics

Study type	***N***	%
RCT	50	10.80
Prospective observational	195	42.12
Retrospective observational	117	25.27
Narrative or systematic review, meta-analysis	49	10.58
Survey	12	2.59
Case report	39	8.64
**Topic**	***N***	**%**
OHRQOL/esthetics/practice management/socio-demographics	62	13.39
Biomaterials	33	7.13
Diagnosis	87	18.79
Treatment	127	27.43
Growth	55	11.88
New technologies	41	8.86
Periodontics/caries prevention	19	4.10
Side effects	26	5.62
Other	13	2.81
**Number of authors**	***N***	**%**
1–3	111	23.97
4–5	167	36.07
> 5	185	39.96
**Self-citation**	***N***	**%**
Yes	274	59.18
No	189	40.82
**Citation metrics**	**Mean (SD)**	**Median (IQR)**
Self-citations	1.67 (6.19)	1 (2)
Total citations	32.01 (18.01)	30 (13)
SCR	5.71 (7.97)	3.03 (8.33)

*SD* standard deviation, *IQR* interquartile range

In 352 out of 463 screened articles, there were at least 4 authors listed. 5.71% of the total citations were counted as self-citations of the first and the last authors with 59.18% of the articles published in 2018 containing at least 1 self-citation (Table 1).

Regarding author rank, last authors self-cited more often than first and first and last authors combined, i.e., 177 vs. 56 and 41 authors, respectively (Table 2). Authors of European origin were the most frequently encountered self-citers followed by Asians, i.e., in 32.19% and 26.61% respectively of the articles. The ratio of males to females among the most self-citing authors was 2.48 to 1, i.e., 166 males to 67 females (Table 2).

**Table 2:** Distribution of the self-citing authors per rank, origin, and gender. The rank “First/Last” refers to articles where an equal number of self-citations were observed for first and last authors

	***N***	%
**Rank**		
First	56	20.44
Last	177	64.60
First/Last	41	14.96
**Origin**		
Asia	62	26.61
Europe	75	32.19
North America	35	15.02
South America	23	9.87
Other	38	16.31
**Gender**		
Male	166	71.24
Female	67	28.77

**Fig. 2:**
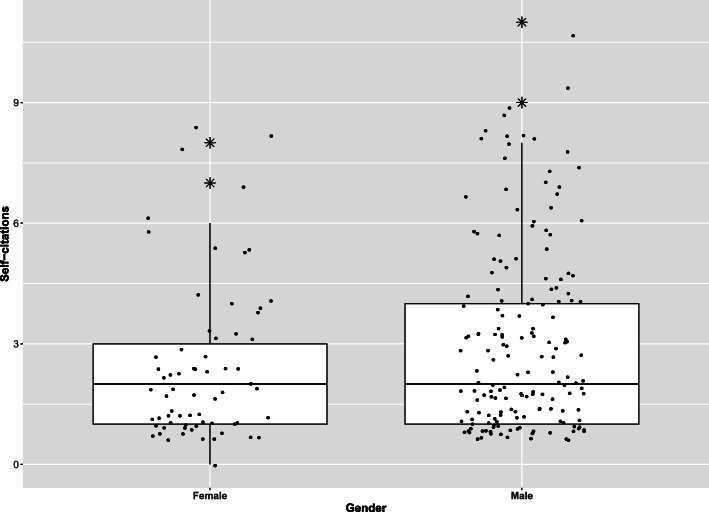
Distribution of self-citations by gender

**Fig. 3:**
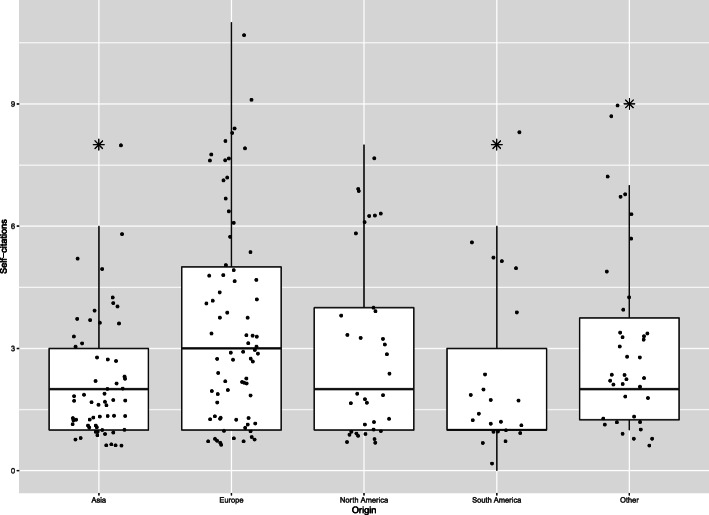
Distribution of self-citations by origin

Boxplots for self-citations per gender, origin, rank, study type, journal, and topic are illustrated in Figs. 2 and 3 and Supplementary Fig. 1, 2. In the univariable analysis, based on the likelihood ratio test, there was no association between self-citation counts and study type (*P* = 0.41), article topic (*P* = 0.61), number of authors (*P* = 0.62), and rank of authors (*P* = 0.56). Author origin (*P* = 0.001), gender (*P* = 0.001), and journal (*P* = 0.05) were associated with self-citation counts, and in the multivariable analysis, only origin and gender remained strong self-citation predictors.

More specifically, self-citations were significantly more common by Europeans and authors originating from affiliations located in Africa, Oceania, or multiple continents (*P*< 0.001 and *P* = 0.03, respectively, Table 3). When comparing genders, male authors cited themselves 1.36 times more than females (*P* ≤ 0.01, Table 3).

**Table 3:** Univariable and multivariable negative binomial regression results

	Univariable			Multivariable		
**Journal**	**IRR**	***P*** **value**	**95% CI**	**IRR**	***P*** **value**	**95% CI**
AO	1.26	0.36	0.76, 2.09	Reference		
AJODO	1.15	0.56	0.72, 1.85	1.10	0.46	0.85, 1.43
OCR	1.10	0.76	0.58, 2.11	1.10	0.65	0.73, 1.67
EJO	1.86	0.02	1.12, 3.10	1.18	0.25	0.89, 1.58
PIOR	1.65	0.09	0.93, 2.91	1.17	0.35	0.84, 1.64
KJO	Reference			1.05	0.82	0.72, 1.53
**Study type**	**IRR**	***P*** **value**	**95% CI**			
RCT	0.73	0.26	0.42, 1.27			
Prospective observational	0.79	0.31	0.51, 1.24			
Retrospective observational	0.62	0.05	0.38, 1.00			
Narrative or systematic review, meta-analysis	0.83	0.52	0.48, 1.45			
Survey	0.61	0.27	0.25, 1.49			
Case report	Reference					
**Topic**	**IRR**	***P*** **value**	**95% CI**			
OHRQOL/esthetics/practice management/socio-demographics	1.79	0.20	0.74, 4.32			
Biomaterials	1.15	0.36	0.60, 3.96			
Diagnosis	1.67	0.25	0.70, 3.95			
Treatment	1.77	0.19	0.76, 4.14			
Growth	2.09	0.10	0.86, 5.07			
New technologies	1.27	0.61	0.50, 3.20			
Periodontics/caries prevention	1.53	0.42	0.55, 4.25			
Side effects	1.23	0.68	0.46, 3.29			
Other	Reference					
**Number of authors**	**IRR**	***P*** **value**	**95% CI**			
1–3	1.17	0.34	0.85, 1.61			
4–5	1.09	0.56	0.82, 1.45			
> 5	Reference					
**Rank**	**IRR**	***P*** **value**	**95% CI**			
First	1.06	0.59	0.86, 1.32			
Last	Reference					
**Origin**	**IRR**	***P*** **value**	**95% CI**	**IRR**	***P*** **value**	**95% CI**
Asia	Reference			Reference		
Europe	1.63	0.00	1.28, 2.08	1.62	< 0.001	1.25, 2.11
North America	1.34	0.06	0.99, 1.82	1.34	0.06	0.99, 1.82
South America	1.06	0.75	0.74, 1.53	1.15	0.46	0.79, 1.67
Other/combination	1.41	0.02	1.05, 1.88	1.41	0.03	1.04, 1.90
**Gender**	**IRR**	***P*** **value**	**95% CI**	**IRR**	***P*** **value**	**95% CI**
Female	Reference			Reference		
Male	1.31	0.01	1.06, 1.62	1.36	< 0.01	1.10, 1.68

*IRR* incidence rate ratio

## Discussion

Bibliometric studies on author self-citation may be helpful in flagging extreme self-promoters and gaming of citation-based indicators in academia. Based on our results, self-citation of authors in orthodontic literature occurs close to previously reported rates in medical specialties [6, 7]. Most importantly, SCR in orthodontics lies below the early estimate of Garfield and Sher [13] or the overall rate of 9% across physical, social sciences, and humanities [14].

Similar to Tolisano et al [7], last authors were more frequent self-citers than first authors, i.e., 1.06 times, but this trend did not reach statistical significance. It is common in scientific writing to name first and last authors in multi-authored scientific papers the most contributed authors. As last authors may be senior researchers holding a high academic rank, self-citation may come naturally in authors with a long track record of publications [15]. First or lead authors, usually early-career researchers, may also have high SCRs because their publications have not been made available long enough to attract other citations [16].

Author origin appears to play an important role in self-referencing as significantly more self-citations were attributed to European authors and authors originating from Africa, Oceania, or multiple affiliations established in different continents compared to Asians. There is evidence that authors from western, individualist cultures are more conducive to self-promoting attitude than authors from more collectivist cultures [15]. Academic promotion policies that require minimum productivity standards might as well explain differences in SCRs between regions [16].

A significant difference in self-citation behavior was observed between male and female authors with men citing themselves 36% more often than women. An overwhelming male dominance in self-citation has been revealed across several disciplines, including biology, sociology, economics, and law, with men self-citing > 50% more often than women over time and up to 70% in recent years [15]. As self-citations do not only directly improve an author’s citation record but also accumulate citations from others in the short term [17], gender discrepancies in SCRs may be further aggravated and have a detrimental effect on scholarly visibility, and likely on academic careers. In case the gender imbalance persists, certain measures need to be introduced by academic administrations to make evaluation processes for hiring and tenures less gender-biased and promote equity in the academic orthodontic community.

Unlike findings in general medical literature [6], the article topic did not have a significant influence on self-citations. Nevertheless, conducting specialized research can be presumed to increase the chances for self-citations. In our sample, the highest SCR was recorded for a case report on CAD-CAM and 3D printing of mini-implant supported orthodontic appliances published in the December issue of AJODO. The last author of this paper cited 7 of his articles in a reference list containing in total 14 citations [18]. With regard to self-citation counts, a record of 19 self-citations was assigned to a user’s guide on cervical vertebral maturation method with both authors being exceptionally productive in publishing on skeletal age assessment [19]. Thus, in cases where authorship is coupled with expertise on innovative research, author self-citation may be anticipated and ethically justified.

There are several limitations to this study that need to be mentioned. First, the selected English orthodontic journals, though high-profile according to JCR ranking, whatever that implies for the submission behavior of authors and the research quality, do not represent the whole orthodontic literature. Despite the main bulk of orthodontic research is getting published in orthodontic journals [20], the dominance of author-level metrics and “publish-or-perish culture” [21] in today’s competitive academic environment forces orthodontic academics to submit to non-orthodontic journals with higher impact factors. Less prestigious medical journals are likely to present higher self-citation percentages [22]. The language in which an article is written influences the odds of receiving citations and deserves attention in bibliometric analyses [23]. Second, this study measured the prevalence of self-citation without assessing the context itself. Quantitative bibliometric research cannot differentiate between legitimate and redundant references to author’s publications. To tackle this issue, meticulous text analyses and discourse-based interviews with the authors have been suggested instead [24].

To the best of our knowledge, this is the first investigation of synchronous author self-citation in a dental subfield. Broad inclusion of journals and broad time-frame used for data collection can be considered strengths of the study design. Whereas previous research was restricted to a few journals and months [6, 7], the current study compiled a full publication year of 6 top orthodontic journals.

More bibliometric research is recommended to describe thoroughly author self-citation in orthodontics and in relation to other subfields in dentistry. Future studies should examine synchronous and diachronous SCRs and patterns over a longer observation period and across a wider range English and non-English orthodontic and dental journals, with and without IF, indexed by various databases. Last but not least, in view of the implications of publication metrics on faculty hiring and promotion, the existing gender difference in author self-citation warrants further investigation to shed light on a possible gender gap in academic hiring, tenure and salary decisions in orthodontic faculties.

## Conclusion

Self-citation practices of first and last authors in orthodontic journals may be considered comparable to those in medical specialties. Author origin and gender seem to be associated with self-citations and should be therefore taken into account when evaluating authors’ attitudes towards self-citation as well as research performance.

## Supplementary Information

**Additional file 1: Figure S1.** Distribution of self-citations by rank, study type, journal, and number of authors.

**Additional file 2: Figure S2.** Distribution of self-citations by topic.

## Data Availability

The datasets used and/or analyzed during the current study are available from the corresponding author on reasonable request.

## Abbreviations

IF: Impact factor
JCR: Journal Citations Reports
OCR: Orthodontics & Craniofacial research
EJO: European Journal of Orthodontics
AJODO: American Journal of Orthodontics and Dentofacial Orthopedics
KJO: The Korean Journal of Orthodontics
AO: The Angle Orthodontist
PIOR: Progress in Orthodontics
OHRQOL: Oral health-related quality of life
SCR: Self-citation rate
