# Supplementary figures and images for: Author self-citation in orthodontics is associated with author origin and gender
Source: Prog Orthod. 2021 Jan 7;22:1. doi: 10.1186/s40510-020-00348-y (PMC7788150; doi:10.1186/s40510-020-00348-y)

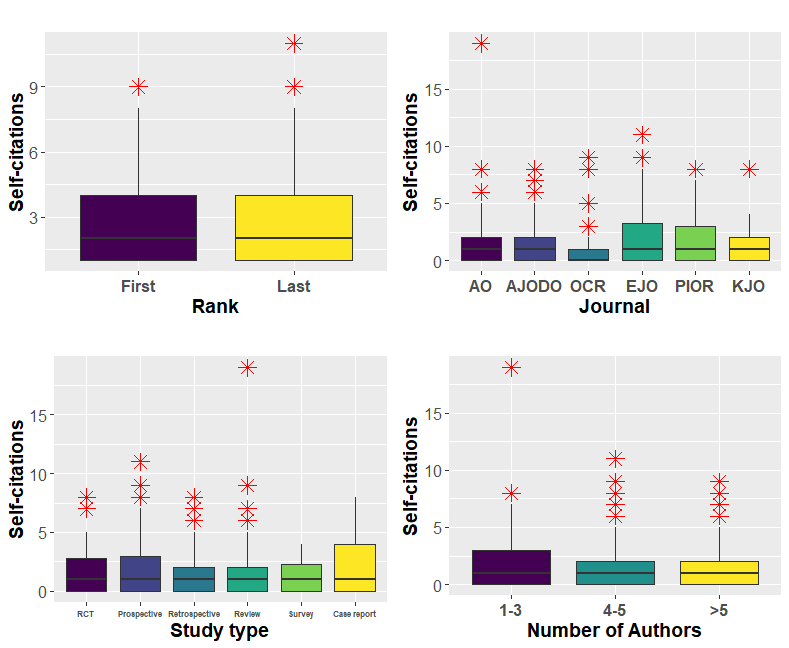

Supplement: Supplementary file 1 — Additional file 1: Figure S1. Distribution of self-citations by rank, study type, journal, and number of authors. [file 40510_2020_348_MOESM1_ESM.png]

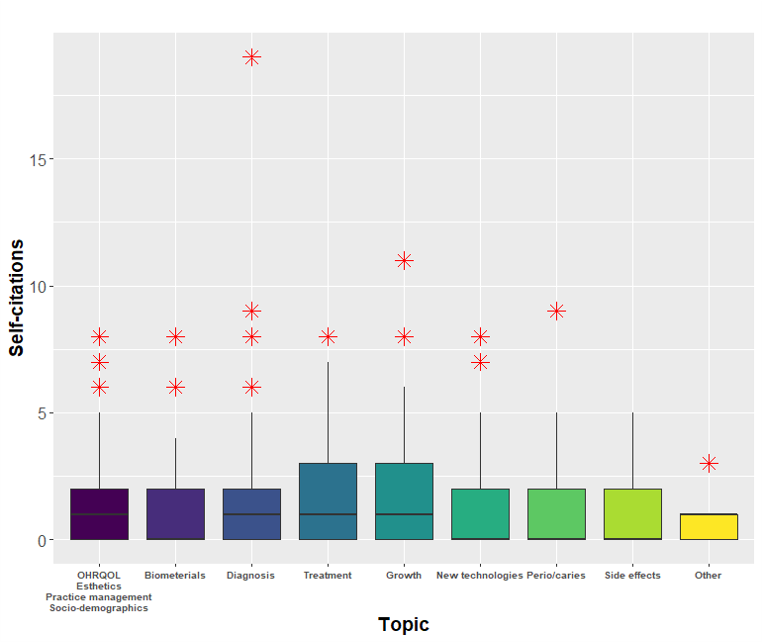

Supplement: Supplementary file 2 — Additional file 2: Figure S2. Distribution of self-citations by topic. [file 40510_2020_348_MOESM2_ESM.png]
